# Hydrothermal Carbon Coating of an Activated Carbon—A New Adsorbent

**DOI:** 10.3390/molecules28124769

**Published:** 2023-06-14

**Authors:** Marta Adame-Pereira, Carlos J. Durán-Valle, Carmen Fernández-González

**Affiliations:** 1 Departamento de Química Orgánica e Inorgánica, Universidad de Extremadura, Avda. de Elvas, s/n, 06006 Badajoz, Spain; martaap@unex.es (M.A.-P.); mcfernan@unex.es (C.F.-G.); 2 IACYS, Universidad de Extremadura, Avda. de Elvas, s/n, 06006 Badajoz, Spain

**Keywords:** acetamiprid, adsorption, activated carbon, hydrothermal carbon, pesticide, water remediation

## Abstract

A new adsorbent material was prepared by coating an activated carbon with hydrothermal carbon obtained from sucrose. The material obtained has different properties from the sum of the properties of the activated carbon and the hydrothermal carbon, which shows that a new material was obtained. It has a high specific surface area (1051.9 m^2^ g^−1^) and is slightly more acidic than the starting activated carbon (p.z.c.-point of zero charge 8.71 vs. 9.09). The adsorptive properties of a commercial carbon (Norit RX-3 Extra) were improved over a wide pH and temperature range. The capacity values of the monolayer according to Langmuir’s model reached 588 mg g^−1^ for the commercial product and 769 mg g^−1^ for the new adsorbent.

## 1. Introduction

Carbonaceous materials present a large number of physical forms at the macroscopic (granular, monoliths, fibres, fabrics, etc.) and microscopic (fullerenes, graphene, nanotubes, etc.) scale [1,2]. One of the classic and best-known carbonaceous materials is activated carbon [3,4,5]. It is usually obtained in a double carbonisation and activation process at high temperature. This material is characterised [6,7] by a highly developed porous structure, a generally alkaline character and a predominantly hydrophobic surface. These properties can be modified by appropriate chemical or thermal treatment, as well as by changing the raw material or preparation method, as is well known [8,9,10,11].

Another more recently studied carbonaceous material is hydrothermal carbon [12,13]. It is obtained by treating carbon-rich materials at a moderate temperature with water in a closed vessel to ensure that the endogenous water vapour pressure acts on this material. Generally, some type of biomass is used as raw material. It is characterised by low pore development, acidity and a hydrophilic surface with a large number of surface groups. As in the previous case, these properties can be modified by thermal or chemical treatments [14].

In this work, we considered whether it was possible to obtain the combined properties of both materials to develop a better adsorbent. Our aim was to achieve a high surface area and polar adsorbent in an environmentally friendly way. Attempts to achieve this goal using hard templates have been reported in previous studies [14,15]. We decided to follow the strategy of coating the surface of an activated carbon with a hydrothermal carbon generated in situ.

Hydrothermal carbon coatings have been previously used on other materials, such as ZnO [16], TiO_2_ [17], sepiolite [18] and Fe_2_O_3_ [19], although there are few examples of coating a carbonaceous material with hydrothermal carbon. In one such case [15], carbon microfibres were coated with hydrothermal carbon and then sulphonated to use this material as an acid catalyst. A paper on coating carbon fibres with hydrothermal carbon has also been published [20]. However, to our knowledge, this has not been attempted on activated carbon.

We tested these hybrid carbonaceous materials on the adsorption of acetamiprid. The aim was to improve the adsorption capacity of a commercial activated carbon, as this method could be a simple, economical and environmentally friendly way to improve this industrial product. To this end, we prepared a hydrothermal carbon from a sucrose solution in a container where activated carbon had been introduced. The result, although positive, was not exactly as expected.

Acetamiprid ((E)-N1-[(6-chloro-3-pyridyl)methyl]-N2-cyano-N1-methylacetamidine) is a neonicotinoid insecticide [21] that replaces the more toxic imidacloprid. It is water soluble (2.950 g L^−1^), and although it is not persistent in soil systems, it can be persistent in aquatic systems. It is highly toxic to birds and earthworms and has medium toxicity to aquatic organisms and mammals. In addition, it has a high accumulation potential [22]. Due to its relative novelty on the market, there are not many references of its removal. Photocatalysis [23,24], adsorption [25,26,27], ozonation [28], wetland [29] and white-rot fungus [30] have been used for this purpose.

## 2. Results and Discussion

### 2.1. Chemical Characterisation of Adsorbents

#### 2.1.1. Composition

The data obtained from the elemental analysis (Table 1) indicate that, in general, these carbons have a high carbon content, followed by a high oxygen content. The carbon prepared from sucrose (CHT) was the one with the lowest carbon content and the highest H and O content, highlighting the almost null N and S content, as expected. The hydrothermal process carried out on a commercial carbon Norit RX-3 Extra (CA) gave rise to a carbon (CAHT) with higher C and N content and a decrease in oxygen content. On the other hand, hydrothermal carbons prepared from CA and sucrose (CT/CA) show an increase in C and N content and a decrease in H and O content. This was observed in all the hybrid carbons, which implies that the hydrothermal treatment affected the activated carbon composition even if the coating process was not carried out.

In general, these carbons have a high fixed carbon content, which makes them promising as adsorbent materials (Table 2). The CHT carbon has high volatile and low carbon content, as well as zero-ash content, as expected given the raw material used. The hydrothermal treatment of CA with water (CAHT) reduced the amount of fixed carbon and ash, increasing the amount of volatiles. The hydrothermal treatment with sucrose solution (CT/CA13) further reduced the ash content. It is important to highlight the low ash content of these materials, as it is convenient that this content is small in the resulting adsorbent; this prevents the possible undesirable effects that the inorganic matter present in the adsorbent may have on certain catalytic and adsorption processes and thus on the possible applications of the material.

**Figure 1:**
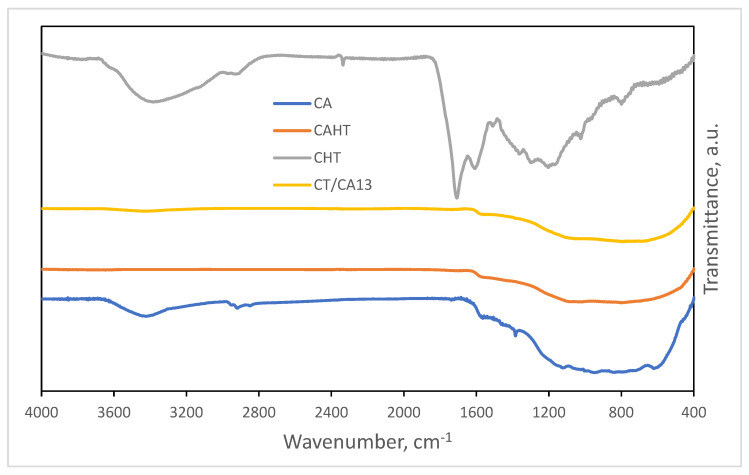
FTIR spectra. Samples: CA, CAHT, CHT and CT/CA13.

The IR spectra recorded for the carbons studied in this work are shown in Figure 1. They show a series of absorption bands whose intensities depend on each carbon. The IR spectrum of CA carbon presents an intensity band at 3400 cm^−1^, which can be associated with the ν(O-H) in hydroxyl groups. The band at 1722 cm^−1^ can be attributed to the stress vibration of the ν(C=O) bond in carboxylic acid groups, while the bands between 1300 and 1000 cm^−1^ can be assigned to the ν(C-O) in different atomic groupings, such as ether-like structures. The lower intensity bands centred at 879 and 687 cm^−1^ are compatible with the existence of substitution in the aromatic rings.

The hydrothermal treatment (CAHT) and the treatment with sucrose to the CA gave rise to similar spectra, and the intensity of the bands decreased, indicating that the aforementioned functional groups and structures must be found in a lower concentration.

The IR spectrum corresponding to the CHT sample presents some variations in the bands with respect to the rest of the samples, which agrees with its chemical nature. The associated band above 3400 cm^−1^ corresponds to the ν(O-H) in hydroxyl groups [31]. The band above 1700 cm^−1^ may be attributable to the C=O vibration of carboxyl groups, while the bands in the region of 1000–1450 cm^−1^ correspond to ν(C-O) (hydroxyl, ester or ether groups) stretching and O-H bending vibrations [32,33,34]. The bands at 875–705 cm^−1^ are assigned to aromatic C-H out-of-plane bending vibrations [35].

#### 2.1.2. Acidic and Basic Properties

The p.z.c. values (Table 3) and the amount of acidic and basic groups also show that this parameter is highly dependent on carbon.

CA is slightly basic, and sucrose hydrothermal carbon is strongly acidic, as expected. Hydrothermal water treatment (CAHT) produces higher alkalinity, increases the number of acidic functional groups and decreases the number of alkaline ones. As for the hybrid carbon (CT/CA13), the hydrothermal treatment reduced its alkalinity, although in small proportion, and increased the number of acidic functional groups.

The acidic and basic groups of the adsorbents were also measured by titration. The hydrothermal treatment with sucrose increased the number of acidic functional groups and slightly reduced the number of basic functional groups. As for CHT, the number of acidic groups was higher, as would be expected. However, it was impossible to measure the basic groups under the same conditions as the other carbons, since the solid was more acidic than the nitric acid solution used, thus obtaining a negative result in the titration.

As a summary on the chemical structure of the surface, the hydrothermal treatment reduced the oxygen content of the original activated carbon (13.36% oxygen + ash − 5.19% ash = 8.17% oxygen) by obtaining CAHT (7.08 % oxygen and ash − 4.23% ash = 2.85% oxygen), leaving less functional groups on the surface. However, the use of sucrose to form a hydrothermal carbon coating also increased the oxygen content (8.12% oxygen and ash − 1.96% ash = 6.16% oxygen), which was the aim of this synthesis. In addition, more basic functional groups were degraded (from 0.56 to 0.46 meq g^−1^), and the number of acidic groups increased (from 0.32 to 0.68 meq g^−1^).

### 2.2. Structural Characterisation

#### 2.2.1. N_2_ Adsorption

**Figure 2:**
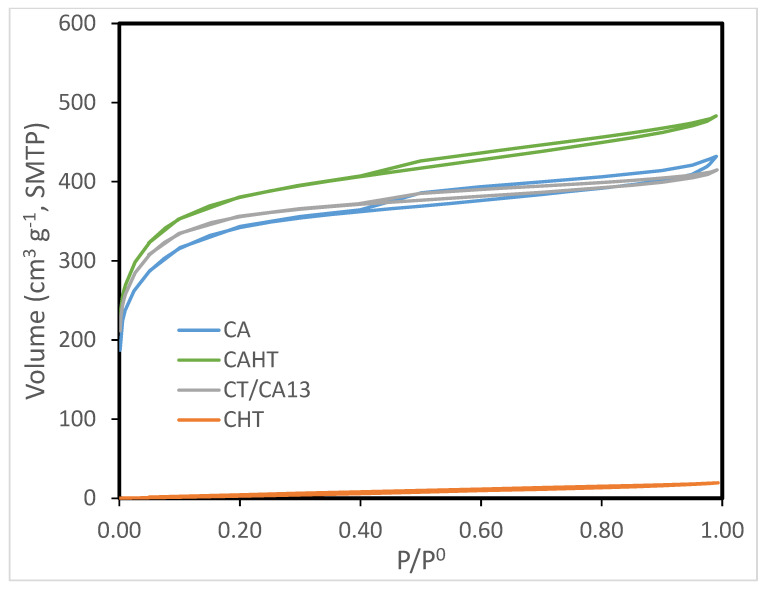
Adsorption isotherms of N2 a −196 °C.

The N_2_ adsorption isotherms for CA, CAHT, CT/CA13 and CHT are plotted in Figure 2. The textural data calculated from these isotherms are included in Table 4. From both the isotherms and the textural data, it is evident that the hydrothermal processes to which CA was subjected (with or without sucrose) did not reduce the porous development of the activated carbon.

The shape of the N_2_ adsorption isotherms (Figure 2) is similar to type IV of the BDDT [36] and the most recent IUPAC [37] classification, thereby indicating that solids contain mesopores. The adsorption follows the monolayer–multilayer–capillary condensation mechanism, i.e., firstly, a monolayer of adsorbate molecules is formed on the surface of the adsorbent solid, and then the process continues with the formation of new layers on top of the first layer. This causes the free release inside the pore to decrease until the phenomenon of capillary condensation occurs at a certain value of the radius. The variation of the adsorbed quantity with increasing equilibrium relative pressure (P/P^0^) indicates that the porosity distribution in the micropore region is wider at CAHT > CT/CA13 > CA. In the mesopore region, the porosity distribution follows the order CAHT > CA > CT/CA13. The increase in the amount adsorbed with P/P^0^ indicates that these carbons contain not only a high volume of micropores but also mesopores of different sizes (Table 4). The extension of the mesopores hysteresis zone up to high-pressure values indicates that slit-shaped pores predominate.

The adsorption isotherm determined for CHT shows that the N_2_ adsorption capacity is particularly low, denoting poor development of micro- and mesoporosity.

#### 2.2.2. Scanning Electron Microscopy

**Figure 3:**
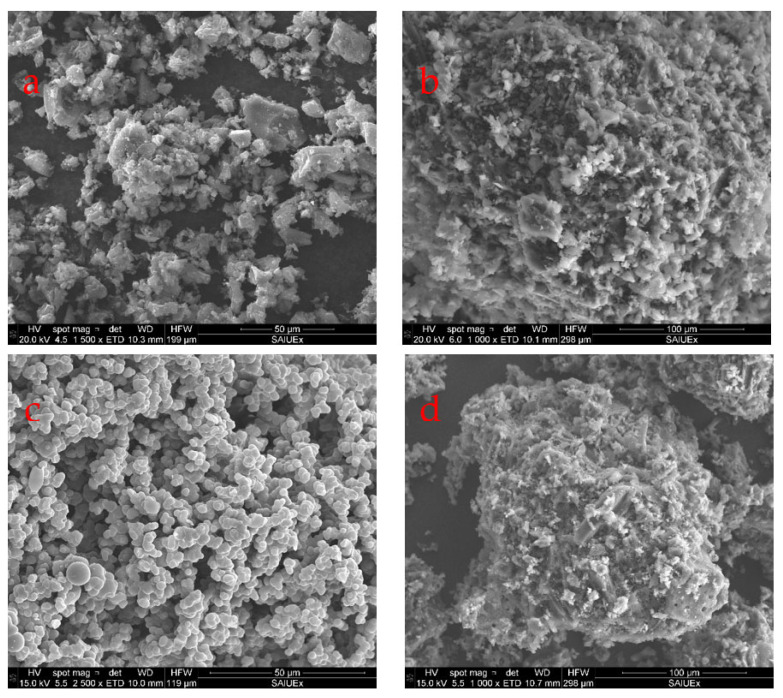
SEM images of (**a**) CA; (**b**) CAHT; (**c**) CHT; and (**d**) CT/CA13.

Some representative images obtained by SEM are shown in Figure 3.

The typical spheres produced when obtaining hydrothermal carbon from saccharides can be seen in Figure 3c (CHT) but not in Figure 3d (CT/CA13). This confirms that the hydrothermal carbon did not precipitate separately from the activated carbon but became integrated and coated with it.

### 2.3. Acetamiprid Adsorption

#### 2.3.1. Adsorbent Selection

Coated carbons were tested in acetamiprid removal, and the discussion on selecting the best adsorbent can be found in the Supplementary Materials. As a summary of this discussion, it can be stated that results suggest that the good properties of CT/CA13 are not due to the presence of the hydrothermal carbon or the activated carbon but to a synergy of both, since the overall result is better than that of the CAHT and CHT materials separately. Therefore, the CT/CA13 carbon was selected for further analysis and the CA and CHT carbons for comparison purposes.

#### 2.3.2. Adsorption Kinetics

**Figure 4:**
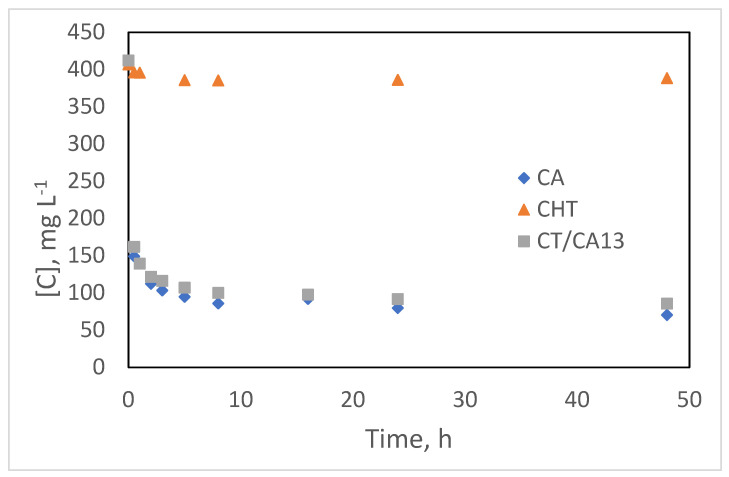
Adsorption kinetics of acetamiprid on the adsorbents prepared.

Figure 4 shows the graph of concentration (mg L^−1^) versus time (h) obtained for the adsorption of acetamiprid with the selected samples. Firstly, it can be seen from this plot that for all samples, most of the acetamiprid was adsorbed at contact times ≤ 8 h. Secondly, it should be noted that the amount of acetamiprid adsorbed with respect to the initial concentration was similar for CA and CT/CA13 (79% and 75%, respectively) and low for CHT (5%). The behaviour of these samples with respect to acetamiprid adsorption is in agreement with the development of microporosity. Table 5 shows that V_DR_ was significantly higher for CA and CT/CA13 than for CHT. In fact, it is well known that the active centres for adsorption are preferentially located in the micropores. Moreover, it should be noted that the small variations obtained in the adsorption of acetamiprid by CA and CT/CA13 with respect to the data obtained in previous adsorption tests could be due to the heterogeneous surface of the samples and the larger size of the micropores of CA with respect to CT/CA13 (Table 4), which would facilitate a greater entry of the adsorbate.

The fit of the kinetic data to the pseudo-first-order and pseudo-second-order models is shown in Table 5. From the R^2^ values, it is observed that the kinetic data fit the pseudo-second-order model better than the pseudo-first-order model. The calculated value of q_e_ in the pseudo-second-order model is closer to that observed experimentally, which supports its validity. Lastly, the k_2_ constant varied as CHT >> CT/CA13 > CA, indicating a higher adsorption rate.

#### 2.3.3. Adsorption Isotherms

**Figure 5:**
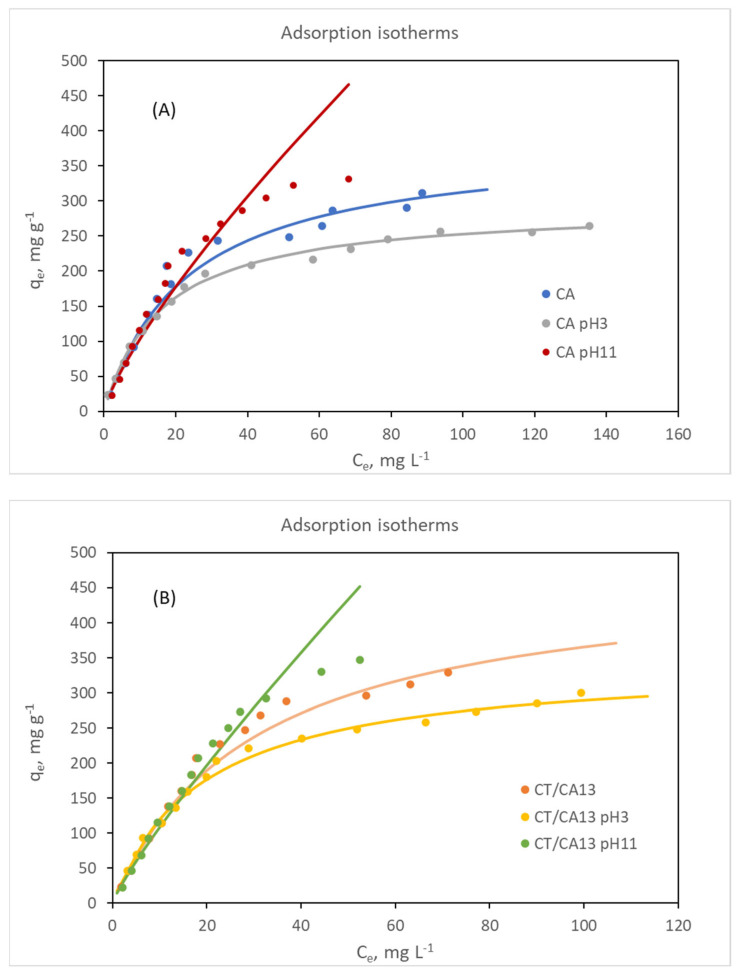
Adsorption isotherms of acetamiprid for CA (**A**) and CT/CA13 (**B**) at different pH values (natural 6.1, 3 and 11): The solid lines represent the Langmuir (natural pH and 3) and Freundlich (pH 11) equations.

The adsorption isotherms obtained for the samples and acetamiprid in aqueous solution are represented in Figure 5. This graph shows that for all cases under study, adsorption was higher with the CT/CA13-coated carbon than with CA and that adsorption was enhanced by increasing the pH of the acetamiprid solution. According to the Giles classification system of adsorption systems in solution [38], the isotherm shapes resemble the L1-type curve, indicating that as adsorption progresses, fewer adsorption sites are available for the adsorbate. In addition, there is no competition between the adsorbate and the solvent for the adsorption centres. Moreover, the negative or positive charge of the adsorbate surface (pH_s_), the average value of which is given by the p.z.c., must also be considered. When the pH is higher than the p.z.c., the carbons are negatively charged, while the opposite happens when the pH is lower than the p.z.c., with positively charged carbons. Furthermore, from a pH value of 6.1 in the acetamiprid solution and a p.z.c. of 8.7 and 9.1 for CT/CA13 and CA, respectively, it can be considered that slight changes will take place on the surface of the carbons upon contact with the adsorbate solution. On the other hand, the pH of the acetamiprid solution after the adsorption process increased only slightly (pH = 6.1) with respect to distilled water (pH = 5.8). A more detailed discussion on the influence of pH can be found in the following section.

The values resulting from fitting the experimental data to the Langmuir and Freundlich isotherm models are given in Table 6. From the values of R^2^, the adsorption isotherm data satisfactorily fit the Langmuir model for CT/CA13 and CA at pH = 3 and at the pH of the solution. However, for pH = 11, where the best adsorption results were obtained, the data fit better to the Freundlich model.

Observing the capacity of the monolayer (S_m_), in all cases the hydrothermal treatment of activated carbon (CA) with sucrose solution increased the value of this capacity by 17% (pH = 3), 24% (normal pH) and 31% (pH = 11).

#### 2.3.4. Influence of pH

**Figure 6:**
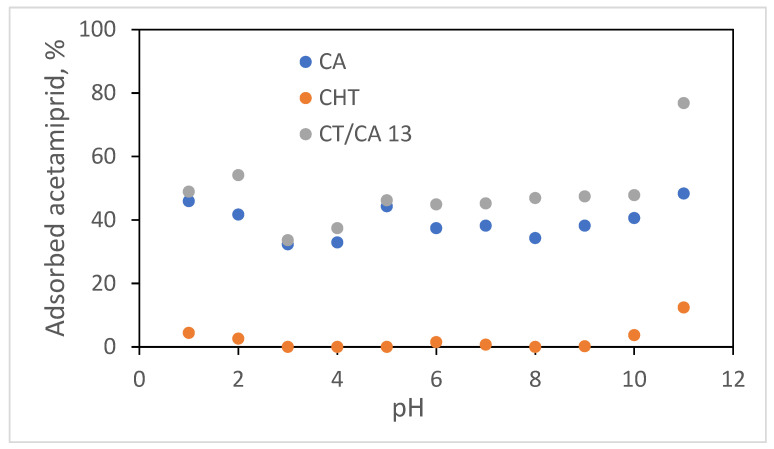
Adsorbed percent of acetamiprid as a function of pH.

The amount of acetamiprid removed as a function of pH is shown in Figure 6.

**Figure 7:**
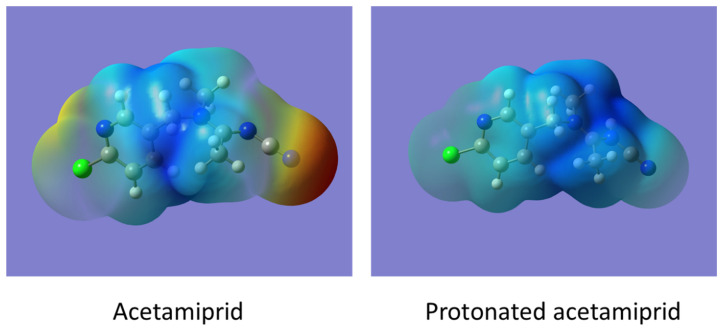
Electron density of acetamiprid and protonated acetamiprid: red colour: negative charge; blue colour: positive charge. Calculated by DFT method.

The pH had little effect on the adsorption capacity of acetamiprid. It was somewhat higher at extremely acidic and especially alkaline pH. This is most clearly observed for the CHT carbon. One possible explanation is that as a medium-sized molecule, there are areas of positive and negative charge on it (Figure 7 left), and when the adsorbent surface has a high charge density (positive at low pH and negative at high pH), this favours adsorption. Between pH 2 and 5, there is a decrease in the amount adsorbed on the CA and CT/CA13 adsorbents. This may be because at these pHs, taking into account the calculated pKa of acetamiprid (4.16) [39], the pesticide molecule becomes positively charged when it picks up a proton from the medium (Figure 7 right) and can thus be repelled by the predominant positive charge on the surface of these two adsorbents. In most situations, the adsorption capacity of CT/CA13 is higher than that of commercial activated carbon. This result indicates that the behaviour of CT/CA13 cannot be explained as a composite of an activated carbon and a hydrothermal carbon, but it constitutes a structure of its own, different from the sum of the two.

#### 2.3.5. Influence of Temperature

**Figure 8:**
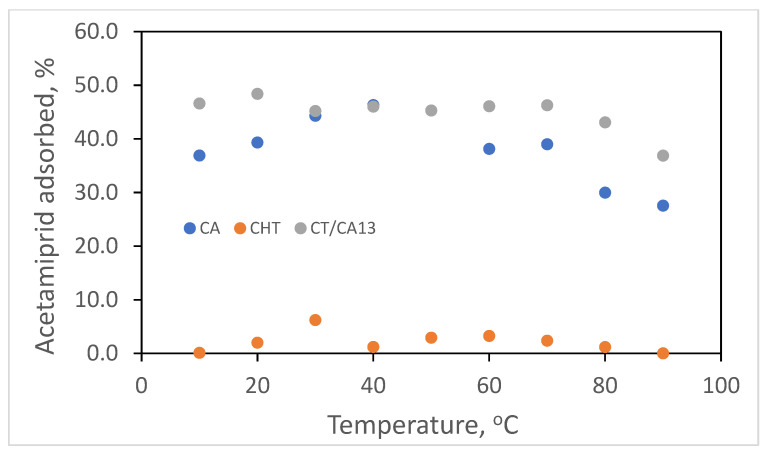
Influence of temperature on acetamiprid adsorption for selected samples.

Figure 8 shows the amount of acetamiprid adsorbed as a function of temperature for the selected samples. For CA, slight increases in the amount of acetamiprid retained at temperatures of 40 °C and 50 °C were obtained (from 44.3% at 30 °C to 46.3% and 45.3% at 40 °C and 50 °C, respectively). For CHT, at the different temperatures, the amount of adsorbate retained was not improved compared with the initial experimental conditions (6.2% at 30 °C). With CT/CA13, the adsorbed amount varied slightly from 10 to 70 °C, with the best results obtained at 20 °C (48.4% at 20 °C). In all cases, adsorption decreased at elevated temperatures (≥70 °C). This may be because, in general, the adsorption process is considered exothermic, and the water solubility of a compound usually increases when the temperature increases [40]. For acetamiprid, the solubility in water increases from 2950 mg L^−1^ at 20 °C to 4250 mg L^−1^ at 25 °C [41]. As in the previous cases, the behaviour of CT/CA13 cannot be explained by the sum of the behaviour of the activated carbon part and the hydrothermal carbon part, but it constitutes a material that differs from the sum of both.

#### 2.3.6. Mechanism

Carbonaceous materials such as those described here usually have a heterogeneous surface with functional groups of varying acidity or alkalinity. This variation can be greater or lesser and can be related to the isotherm model. If there is little variation between the active sites, a better fit to the Langmuir model will be obtained, and if there is greater variety in the active sites (greater heterogeneity), it will be to the Freundlich model. It can also be assumed that as the pH of the solution changes, the surface charge will gradually change, being mainly positive at acidic pH and negative at basic pH. A higher amount of charge (positive or negative) attracts a polar molecule such as acetamiprid more strongly than a simple van der Waals bond between neutral species. This would explain the higher amount adsorbed at extreme pHs.

**Scheme 1:**
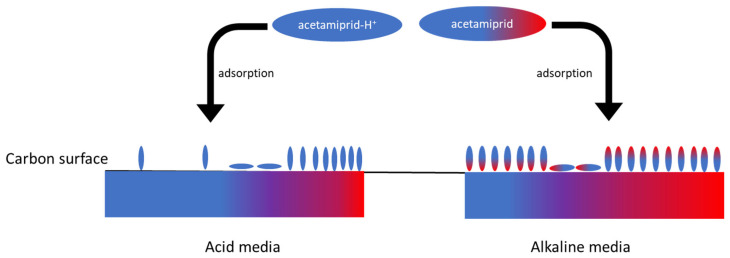
Mechanism of adsorption in acid and alkaline media: blue: positive charge; red, negative charge; and violet, neutral.

It is also possible to consider that both the adsorbate and the adsorbent behave as amphoteric. This is supported by the fact that the lowest adsorption occurs at pH 3. In this case, acetamiprid is positively charged (see Figure 7) and can only bind to negative and neutral active sites. At pH 11, the molecule has positively and negatively charged areas and could bind, with proper orientation, to positive, negative or neutral sites, giving a better adsorption result. A graphical representation of the proposed mechanism is shown in Scheme 1.

## 3. Materials and Methods

### 3.1. Raw Material

A commercial activated carbon Norit RX-3 Extra was employed as a starting carbon (hereinafter CA). CA was first milled using a mill (IKA A10 basic) to <0.200 mm particle size. All reagents were of analytical grade or the commercial product: sucrose (commercial product, Hacendado), sodium hydroxide (98.7%, Fisher Scientific S.L., Madrid, Spain), nitric acid (65%, Fisher Scientific), sodium nitrate (99%, Panreac, Madrid, Spain) and acetamiprid (commercial granular product, 20% wt/wt, Kenogard, Barcelona, Spain).

### 3.2. Synthesis of Coated Carbons

In a Teflon vessel reinforced with a duraluminium outer jacket, the corresponding amount of commercial activated carbon (4, 8 or 12 g of CA) and 50 mL of a sucrose solution of known concentration (0.01, 0.05 or 0.10 g L^−1^) were added [29]. The mixture was placed in an oven (Selecta, model 2001244) and subjected to the temperature and time listed in Table 7. Subsequently, the content was filtered in a funnel with a filter plate and washed with 200 mL of distilled water. Then, it was dried in an oven at 110 °C for 24 h. In addition, a sucrose-only charcoal (CHT) was synthesised, and CA was treated by the hydrothermal method with 50 mL of distilled water (without sucrose) to observe if there was any effect on this material (CAHT). The obtained yields were ≈95% for CAHT and 45% for CHT. The yield values were calculated using Equation (1):

(1) $$Yield\;\left(\%\right)=\frac{M_{F}}{M_{0}}\times 100$$

where *M_F_* is the final mass of the product obtained by the hydrothermal method and *M*_0_ is the starting CA mass (or sucrose in CHT).

### 3.3. Coated Carbons Characterisation

An elemental analysis (C, H, N and S) was carried out using the LECO CHNS-932 equipment, and the difference was assigned to oxygen and ash content.

A thermogravimetric analysis was carried out in a thermobalance STA449F3 Jupiter-Netzsch to determine fixed carbon, volatile matter and ash [42,43]. Firstly, the sample was heat-treated from room temperature to 105 °C in an argon atmosphere (gas flow = 100 mL min^−1^) at a rate of 30 °C min^−1^. Then, it was kept at this temperature for 10 min. Subsequently, the heating treatment continued at a rate of 30 °C min^−1^ to 900 °C, keeping this temperature for 7 min to obtain the volatile matter content. The atmosphere was then changed to argon/oxygen (80:20, gas flow = 100 mL min^−1^), keeping the temperature for 40 min to burn the fixed carbon. The ash content was the noncombustible residue.

Scanning electron microscopy (SEM) images were obtained with a Quanta 3D FEG microscope (FEI Company), operating in the high vacuum mode at an accelerating voltage of 0.2 to 30 kV, using a secondary electron detector for high vacuum.

The specific surface area was calculated by applying the BET method (S_BET_) [44], the micropore volume by the Dubinin–Radushkevich equation (V_DR_) [45], and the mesopore volume (V_me_, V_ad_ at p/p^0^ = 0.95 at p/p^0^ = 0.10, V_ad_ = volume adsorbed) and total volume (V_total_, V_ad_ at p/p^0^ = 0.95 × (1.547 × 10^−3^)) of the samples were determined by N_2_ adsorption–desorption isotherms at −196 °C in an Autosorb iQ2-C Series (Quantachrome). Previously, the samples were degassed at 250 °C overnight.

The FT-IR spectra for the samples were recorded on a Bruker FT-IR spectrometer VERTEX 70v, using a KBr disc. The discs were prepared using the ratio sample:KBr equal to 1:250 and then pressing the mixture at 10 tonnes cm^−2^ for 5 min under vacuum. One pure KBr disc of the same mass as the sample disc was also prepared, and its spectrum was used as background. FTIR spectrums were recorded between 4000 and 400 cm^−1^ using 50 scans and a resolution of 0.4 cm^−1^.

The point of zero charge (p.z.c.) values were determined under batch conditions using the method proposed by Nabais and Carrott [46].

The amount of acid and basic groups present in the different samples was determined in duplicate by acid–base titration. To determine the acid groups, 0.15 g of the sample was mixed with 30 mL of 0.01 M NaOH for 24 h at 25 °C in a thermostatic bath with continuous agitation, and 20 mL of the filtrate was titrated with 0.01 M HNO_3_. Conversely, for the determination of the basic groups, 0.15 g of the sample was mixed with 30 mL of 0.01 M HNO_3_ for 24 h at 25 °C, and 20 mL of the filtrate was titrated with 0.01 M NaOH. In the acid-base titration and p.z.c. measurements, a pH meter (Hanna, model HALO HI11312) was used until a value of pH = 7.0 was reached. From the volumes obtained in the titrations, the meq g^−1^ of acidic and basic sites was calculated.

### 3.4. Adsorption of Acetamiprid

In the study of the adsorption process of acetamiprid (M.W. = 222.67 g mol^−1^) in aqueous solution, firstly, the adsorption capacity of this insecticide was tested for the different adsorbents prepared in this work. All adsorption studies were carried out in triplicate in a thermostatic bath at 30 °C for 24 h at constant stirring by the batch procedure using an adsorbent amount of 10 mg and 40 mL of a 200 mg L^−1^ aqueous solution of acetamiprid (pH ≈ 5.8). Previously, the UV–Vis spectrum was recorded in a Shimadzu UV-1800 spectrophotometer using quartz cuvettes of 1 cm optical path. The wavelength corresponding to the maximum absorbance was obtained at 243 nm [47]. In addition, the chemical stability of acetamiprid in aqueous solution was studied. To this end, no changes in colour or absorbance of the solution were observed after two weeks. The adsorption capacity of acetamiprid was quantified using the mass balance Equation (2):

(2) $$Q_{e}=\left(\frac{C_{0}-C_{F}}{W}\right)\times V$$

where *Q_e_* is the amount of acetamiprid adsorbed per gram of adsorbent (mg g^−1^), *C*_0_ and *C_F_* are the initial and final acetamiprid concentrations (mg L^−1^) in the aqueous solutions, respectively, *W* is the adsorbent mass (g) and *V* is the volume of acetamiprid solution (L). The results of the adsorption capacity were used to identify the best preparation conditions for hybrid carbon. The best adsorbent and CA and CHT carbons (selected for comparison purposes) were utilised in the subsequent studies of the adsorption capacity of acetamiprid at different pH values (ranging from 1 to 11) to investigate the influence of pH and temperature (ranging from 10 °C to 90 °C) on the adsorption kinetics and equilibrium of acetamiprid.

The adsorption kinetics analyses were performed in duplicate by the batch procedure under constant stirring at 30 °C in a thermostatic bath using 200 mg of adsorbent and 200 mL of a 400 mg L^−1^ aqueous solution of acetamiprid. The criterion for defining the equilibrium time was that the adsorption rate should be less than 1% of the initial rate. The adsorption isotherms were measured by the batch procedure using 40 mg of adsorbent and 40 mL of acetamiprid in aqueous solution of different concentrations (from 25 to 1000 mg L^−1^ of acetamiprid) at 30 °C and continuous stirring. To ensure that equilibrium was achieved, the aliquots to be analysed were extracted at 24 h.

The kinetic data were fitted to the pseudo-first-order kinetic [48] and pseudo-second-order kinetic [49] models. Lagergren’s equation rearranged to a linear form is usually written as Equation (3):

(3) $$log\left(q_{e}-q_{t}\right)=log\;q_{e}-\frac{K_{1}}{2.303}t$$

where *q_e_* and *q_t_* are the amounts adsorbed at equilibrium and at time *t* (mg g^−1^), respectively, and *K*_1_ is the pseudo-first-order rate constant (h^−1^). The representation of *log(q_e_ − q_t_)* versus t should be a straight line with slope log *q_e_* and intercept −*K*_1_/2.303.

The Ho and McKay equation is (Equation (4)):

(4) $$\frac{t}{q_{t}}=\frac{1}{K_{2}q_{e}^{2}}+\frac{1}{q_{e}}t$$

where *q_t_* and *q_e_* are the adsorption capacities at time *t* and at equilibrium, respectively (mg g^−1^), and *K_2_* is the rate constant of pseudo-second-order adsorption (g mg^−1^ h^−1^). In this case, the plot of *t*/*q_t_* versus t should generate a linear relationship, from which *q_e_* and *K*_2_ can be obtained from the slope and the intersection of the plot.

The adsorption isotherms were measured at different conditions of pH (dissolution pH 3 and 11). The pH values 3 and 11 were obtained by citric acid/Na_2_HPO_4_ and glycine/NaOH buffer solutions, respectively. Moreover, the models of Langmuir [50] and Freundlich [51] were applied to the data obtained from the isotherms. Langmuir’s equation can be written as:

(5) $$\frac{C_{e}}{q_{e}}=\frac{1}{S_{m}K_{L}}+\frac{C_{e}}{S_{m}}$$

where *C_e_* (mg L^−1^) and *q_e_* (mg g^−1^) are the equilibrium concentrations in the liquid and adsorbed quantity in the solid phase, respectively. On the other hand, *S_m_* is related to the maximum adsorption capacity, and *K_L_* is the characteristic constant associated with adsorption intensity. The representation of *C_e_*/*q_e_* against *C_e_* generates a straight line; thus, from the slope and the intersection of the graph, 1/*S_m_* and 1/*S_m_K_L_* can be obtained.

Freundlich’s equation is usually represented as Equation (6):

(6) $$log\;q_{e}=log\;K_{F}+\frac{1}{n}log\;C_{e}$$

where *K_F_* and *n* are constants related to adsorption capacity and adsorption intensity. The plot of *log q_e_* versus *C_e_* should generate a linear relationship from which *log K_F_* and 1/*n* can be obtained.

The computational study of the electronic density of acetamiprid was performed using the Gaussian16 package [52] at the M06-2x/6-311G++(d,p) level of theory. The calculations were carried out in water (SMD model) [53].

## 4. Conclusions

Hydrothermal treatment of an activated carbon in the presence of sucrose improves its adsorbent properties. This improvement is based on the coating of the surface of the activated carbon. The result is a material with a slightly lower specific surface area, higher surface acidity and lower mineral matter content. The surface chemical structure changes, reducing the number of basic functional groups and increasing the number of acidic groups. The adsorption capacity improved over a wide range of pH and temperature values. This new adsorbent showed a high capacity to remove acetamiprid from the aqueous solution, which is the environment where this pesticide persists the most easily. These new materials are being tested as adsorbents for various pollutants in our laboratory. However, given their novelty, it is necessary to study the effect of different raw materials for both support and coating. It is also necessary to extend the characterisation techniques of the adsorbents, to study a wider variety of contaminants and to better understand the operation mechanism of these materials.

## Figures and Tables

**Table 1:** Elemental analysis (C, H, N, S and O + ash by difference).

Sample	%C	%H	%N	%S	%(O + Ash)
CA	84.43	1.29	0.47	0.45	13.36
CAHT	89.69	1.63	1.21	0.39	7.08
CT/CA11	90.97	1.20	1.61	0.56	5.66
CT/CA12	90.52	0.93	1.45	0.50	6.60
CT/CA13	89.21	1.05	1.32	0.30	8.12
CT/CA14	91.21	0.80	1.34	0.59	6.06
CT/CA15	91.28	0.49	1.78	0.34	6.11
CT/CA16	91.04	0.77	1.64	0.57	5.98
CT/CA17	89.48	0.80	1.30	0.49	7.93
CT/CA18	89.82	0.70	1.39	0.52	7.57
CT/CA19	90.45	0.68	1.31	0.31	7.25
CHT	64.30	6.66	0.00	0.01	29.03

**Table 2:** Volatile matter, fixed carbon and ash content (dry basis).

	CA	CAHT	CHT	CT/CA13
Volatile	2.46	3.79	50.95	3.93
Fixed carbon	92.36	91.97	49.05	94.11
Ash	5.19	4.23	0.00	1.96

**Table 3:** P.z.c. measurements: acidic and basic functional groups.

	P.z.c.	Acidic Groups meq g^−1^	Basic Groups meq g^−1^
CA	9.09	0.32	0.56
CAHT	8.04	0.51	0.26
CHT	2.77	1.96	---
CT/CA13	8.71	0.68	0.46

**Table 4:** Adsorption isotherms of N_2_ at −196 °C: textural data.

Sample	S_BET_, m^2^ g^−1^	V_DR_, cm^3^ g^−1^	V_me_, cm^3^ g^−1^	R_poro_, nm	V_total_, cm^3^ g^−1^
CA	1025.5	0.498	0.145	0.741	0.668
CAHT	1144.5	0.542	0.182	0.591	0.728
CT/CA13	1051.9	0.516	0.109	0.591	0.628
CHT	27.1 ^1^	0.001	0.025	---	0.027

^1^ The fit of the experimental data to the BET model does not meet the parameters recommended by IUPAC; thus, the estimated value for CHT cannot be considered accurate.

**Table 5:** Fitting of kinetic data of acetamiprid adsorption to pseudo-first-order and pseudo-second-order models.

		CA	CHT	CT/CA13
Pseudo-1st	k_1_	0.0958	0.0088	0.1971
	q_e_ (calc)	72.79	1.41	104.02
	R^2^	0.5452	0.0023	0.903
Pseudo-2nd	k_2_	0.0080	0.1819	0.0104
	q_e_ (calc)	336.70	21.14	326.80
	R^2^	0.9995	0.9981	0.9998

**Table 6:** Fitting of isotherms data of acetamiprid adsorption to the Langmuir and Freundlich models.

	Langmuir Model			Freundlich Model		
pH	Parameter	CA	CT/CA13		CA	CT/CA13
3	S_m_	294.1	344.8	K_F_	31.59	35.20
	K_L_	0.0622	0.0523	n	2.077	2.016
	R^2^	0.9773	0.9956	R^2^	0.9393	0.9347
Normal	S_m_	384.6	476.2	K_F_	24.05	20.00
	K_L_	0.0430	0.0331	n	1.605	1.379
	R^2^	0.9975	0.9573	R^2^	0.9090	0.9437
11	S_m_	588.2	769.2	K_F_	16.89	14.94
	K_L_	0.0239	0.0175	n	1.272	1.161
	R^2^	0.9080	0.8607	R^2^	0.9480	0.9754

**Table 7:** Preparation of the coated carbons and sample code.

Code	CA Mass (g)	Sucrose Concentration (g L^−1^)	Temperature (°C)	Isothermal Time (h)
CA	Carbon Norit RX-3 Extra without treatment			
CAHT	4	-	180	20
CHT	-	20 g dissolved in 50 mL of water (400 g L^−1^)	180	20
CT/CA11	4	0.01	160	10
CT/CA12	4	0.05	180	15
CT/CA13	4	0.10	200	20
CT/CA14	8	0.05	160	20
CT/CA15	8	0.10	180	10
CT/CA16	8	0.01	200	15
CT/CA17	12	0.10	160	15
CT/CA18	12	0.01	180	20
CT/CA19	12	0.05	200	10

## Data Availability

No data available.

## Supplementary Materials

The following supporting information can be downloaded at: https://www.mdpi.com/article/10.3390/molecules28124769/s1, Figure S1: Fits to pseudo-first (left) and pseudo-second (right) order kinetic models; Figure S2: Fit to Langmuir (left) and Freundlich (right) models of isotherm; Table S1: Adsorption capacity of the adsorbent; Table S2: Influence of experimental parameters on the preparation of the hybrid carbons, [54].

## Sample Availability

Samples of the compounds are not available from the authors.
