# Supplementary material for: Hydrothermal Carbon Coating of an Activated Carbon—A New Adsorbent
Source: Molecules. 2023 Jun 14;28(12):4769. doi: 10.3390/molecules28124769 (PMC10303840; doi:10.3390/molecules28124769)
Supplement: Supplementary file 1 [file molecules-28-04769-s001.zip › molecules-2427665-supplementary.pdf]

Hydrothermal carbon coating of an activated carbon. A new adsorbent.

Marta Adame-Pereira[1], Carlos J. Durán-Valle[2*], Carmen Fernández-González[1]

Supplementary material

Adsorbent selection

Coated carbons were tested in acetamiprid removal, and the adsorption capacities are listed in Table S1. The initial activated carbon (CA) showed good adsorption capacity (Q_e = 354.7 mg g^{-1}), which slightly deteriorated with the hydrothermal treatment (CAHT, Q_e = 321.0 mg g^{-1}). The hydrothermal carbon from sucrose (CHT) showed a very low adsorption capacity (Q_e = 49.7 mg g^{-1}). This can be explained by their poor surface development, as shown in Table 5. The hybrid carbons presented values close to that of the original material (Q_e range 318.8 to 400.0 mg g^{-1}); thus, they were selected to analyse CT/CA13 (Q_e = 400 mg g^{-1}, prepared at 200 °C and 20 h). These results suggest that the good properties of CT/CA13 are not due to the presence of the hydrothermal carbon or the activated carbon, but to a synergy of both, since the overall result is better than that of the CAHT and CHT materials separately. Therefore, the CT/CA13 carbon was selected for further analysis and the CA and CHT carbons for comparison purposes.

Table S1. Adsorption capacity of the adsorbent.

**Sample**	**Q_e, mg g^{-1}**
CT/CA11	318.8
CT/CA12	350.0
CT/CA13	400.0
CT/CA14	384.9
CT/CA15	367.9
CT/CA16	333.9
CT/CA17	344.3
CT/CA18	384.9
CT/CA19	362.2
CA	354.7
CHT	49.7
CAHT	321.0

It should be noted that the CT/CA13 carbon had the highest proportion of oxygen in its composition (see Table 2). According to a published studies (Zhao et al., 2016), higher oxygen contents and the presence of a porous material favours adsorption. In addition, the hydrothermal treatment influenced the removal of acetamiprid.

The influence of the different preparation parameters on the adsorbents CT/CA11 to CT/CA19 are shown in Table S2. The parameters used to prepare each sample can be found in Table 7 of the main text.

Table S2. Influence of experimental parameters on the preparation of the hybrid carbons.

Parameter	Value	Mean Q_e, mg g^{-1}	Standard deviation
	4	356.25	
Mass activated carbon (g)	8	362.22	3.98
	12	363.81	
	160	349.32	
Temperature (°C)	180	367.58	9.96
	200	365.38	
	0.01	345.87	
Conc. sucrose (g L^{-1})	0.05	365.69	13.14
	0.10	370.72	
	10	349.64	
Time (h)	15	342.71	25.50
	20	389.93	

The mass of activated carbon had little influence on the adsorption capacity of the carbons. The temperature used appeared to be more important, which must be high, as well as the concentration of the sucrose solution used. However, the experimental parameter that produced the greatest variation in the results was the reaction time. Apparently, a certain amount of heat (time + temperature) was needed for the process to be effective. An appreciable amount of sucrose must also be used for its effect to be noticeable.

The preparation of a new material with the best experimental parameters (12 g of activated carbon, 180 °C, 0.10 g L^{-1} of sucrose solution and 20 hours) allowed obtaining a new carbon (CT/CA20) that gave a worse than expected acetamiprid adsorption result (Q_e = 344.8 mg g^{-1}). This indicates that the hydrothermal coating process must be complex and that there are probably synergistic or antagonistic effects between the experimental conditions. For this reason, sample CT/CA20 was discarded and the best sample obtained (CT/CA13) was used instead.

Kinetic models fits

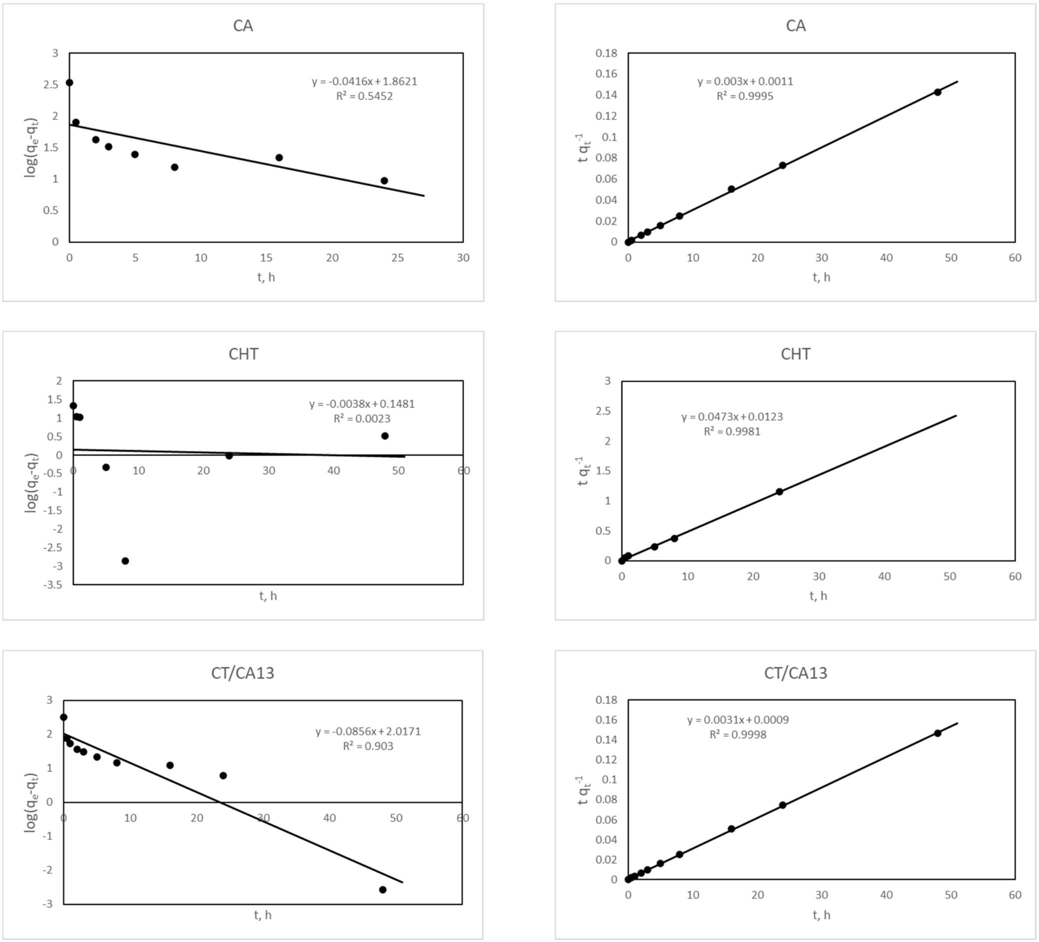

Figure S1. Fits to pseudo-first (left) and pseudo-second (right) order kinetic models.

Isotherm models fit

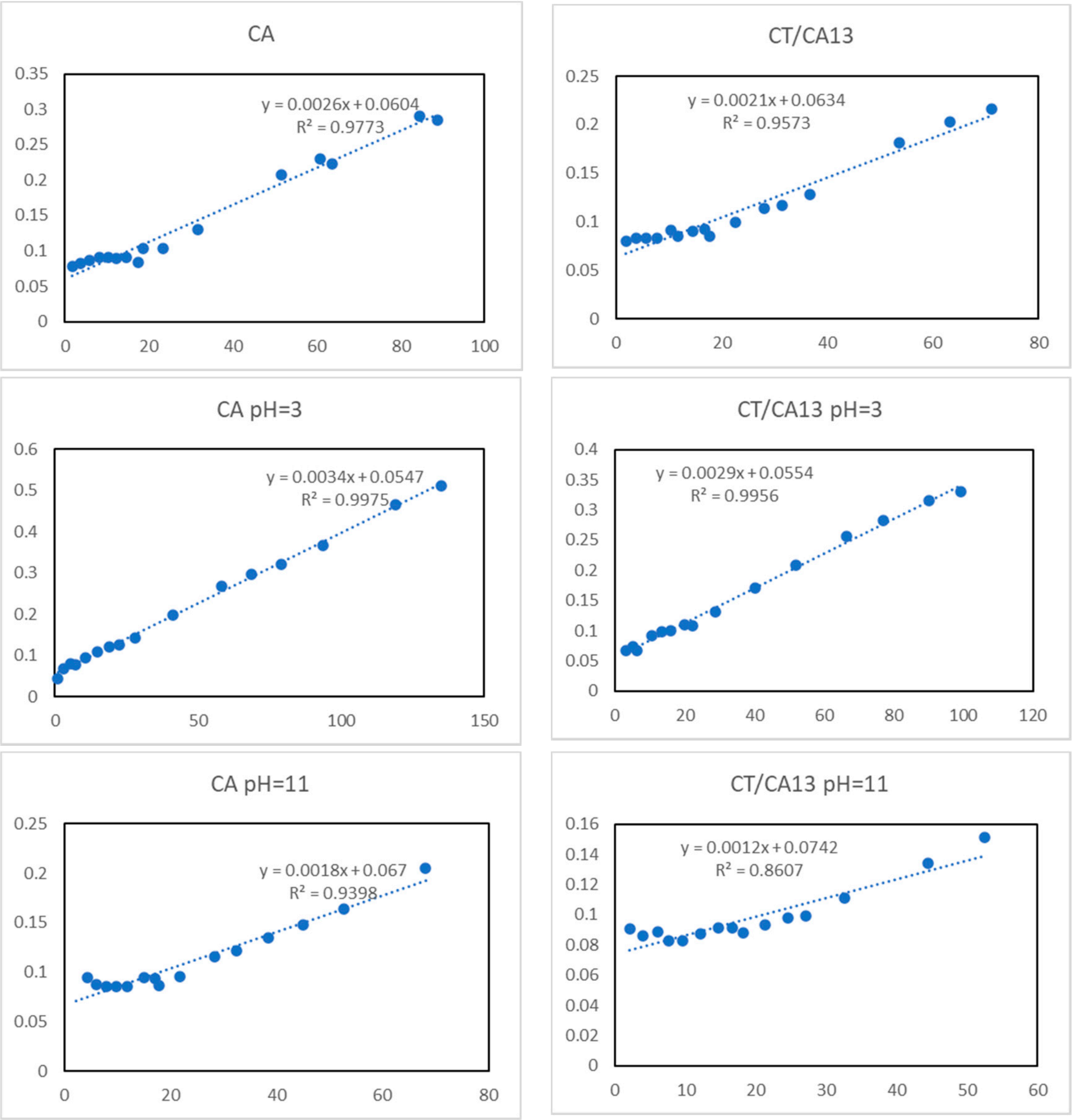

Figure S2. Fit to Langmuir (left) and Freundlich (right) models of isotherm.

Reference

Zhao, R., Wang, Y., Li, X., Sun, B., Li, Y., Ji, H., Qiu, J., & Wang, C. (2016). Surface Activated Hydrothermal Carbon-Coated Electrospun PAN Fiber Membrane with Enhanced Adsorption Properties for Herbicide. *ACS Sustainable Chemistry & Engineering*, *4*, 2584–2592. https://doi.org/10.1021/acssuschemeng.6b00026
